# Nuclear medicine imaging of multiple myeloma, particularly in the relapsed setting

**DOI:** 10.1007/s00259-016-3576-1

**Published:** 2016-11-29

**Authors:** Esther G. M. de Waal, Andor W. J. M. Glaudemans, Carolien P. Schröder, Edo Vellenga, Riemer H. J. A. Slart

**Affiliations:** 1 Department of Hematology, University of Groningen, University Medical Center Groningen, PO Box 30001, 9700 RB Groningen, The Netherlands; 2 Department of Nuclear Medicine and Molecular Imaging, University of Groningen, University Medical Center Groningen, Groningen, The Netherlands; 3 Department of Medical Oncology, University of Groningen, University Medical Center Groningen, Groningen, The Netherlands; 4 Department of Biomedical Photonic Imaging, University of Twente, Enschede, The Netherlands

**Keywords:** Relapsing multiple myeloma, Radiopharmaceutical applications, Nuclear medicine, SPECT, PET, Response monitoring

## Abstract

Multiple myeloma (MM) is characterized by a monoclonal plasma cell population in the bone marrow. Lytic lesions occur in up to 90 % of patients. For many years, whole-body X-ray (WBX) was the method of choice for detecting skeleton abnormalities. However, the value of WBX in relapsing disease is limited because lesions persist post-treatment, which restricts the capacity to distinguish between old, inactive skeletal lesions and new, active ones. Therefore, alternative techniques are necessary to visualize disease activity. Modern imaging techniques such as magnetic resonance imaging, positron emission tomography and computed tomography offer superior detection of myeloma bone disease and extramedullary manifestations. In particular, the properties of nuclear imaging enable the identification of disease activity by directly targeting the specific cellular properties of malignant plasma cells. In this review, an overview is provided of the effectiveness of radiopharmaceuticals that target metabolism, surface receptors and angiogenesis. The available literature data for commonly used nuclear imaging tracers, the promising first results of new tracers, and our pilot work indicate that a number of these radiopharmaceutical applications can be used effectively for staging and response monitoring of relapsing MM patients. Moreover, some tracers can potentially be used for radio immunotherapy.

## Introduction

### Multiple myeloma, diagnosis and treatment

Multiple myeloma (MM) is a disease characterized by a monoclonal plasma cell population in bone marrow. In most cases, the diagnosis of MM is based on the presence of a monoclonal M-protein or free-light chain in the blood and at least 10 % plasma cells in the bone marrow. Treatment is initiated when MM is symptomatic according to CRAB features: hypercalcemia (C), renal impairment (R), anemia (A) or bone lesions (B). Currently, patients with more than 60 % monoclonal plasma cells in their bone marrow, a free-light chain ratio greater than 100, or more than 1 focal bone or bone marrow lesion on the magnetic resonance imaging (MRI) are classified as high risk for development of MM, and should also receive treatment [1].

First-line treatment for patients younger than 70 years and eligible for autologous stem cell transplantation (ASCT) consists of induction chemotherapy, including a proteasoom inhibitor or an immunomodulator agent like bortezomib, lenalidomide or thalidomide, followed by ASCT [2, 3]. Patients ineligible for ASCT are treated with a combination of melphalan, prednisolone and a novel agent, or with the combination of lenalidomide and dexamethasone [4, 5]. Following the introduction of these regimens, the overall survival (OS) improved considerably: the 5-year OS for younger MM patients is now 70 %, and for older patients, 41 % [6].

### Multiple myeloma and imaging

#### Whole-body X-ray, MRI and low-dose CT scan

Asymptomatic MM is distinguished from symptomatic MM through the CRAB criteria. Bone lesions play an important role since lytic bone lesions develop in 90 % of the patients during the disease. These lesions, are an important cause of morbidity, resulting in pain and, in some cases, in pathologic fractures [7]. Lytic bone lesions are the result of increased bone resorption and reduced bone formation [8]. Detecting bone lesions is an important part for the diagnosis of symptomatic MM since having bone lesions means treatment is indicated. This highlights the need for accurate investigation of bone disease. Until recently, whole-body X-ray (WBX) was the method of choice. This technique has several limitations: it can only detect lesions that have lost more than 30 % of the trabecular bone [7], and no extramedullary disease can be shown. The value in relapsing disease is limited since lesions persist post-treatment. No distinction can be made between old vs. new lesions and, therefore, it is of limited value for disease monitoring.

In recent years, alternative techniques have been developed to visualize MM activity. Low-dose whole-body computed tomography (WB-CT), MRI and positron emission tomography (PET/CT) are introduced for the detection of (active) bone lesions. The latest update from the International Myeloma Working Group defines that for the diagnosis of symptomatic MM, evidence of one or more (>5 mm in size) osteolytic bone destruction lesions seen on CT or PET/CT does fulfill the criteria for bone disease, thereby fulfilling the CRAB criteria [1]. The guideline also recommends performing a PET/CT, CT or MRI of the whole body or spine in all patients suspected of asymptomatic MM to exclude bone involvement [1]. Nowadays, MRI and WB-CT scanning have been implemented in many parts of the world for detecting myeloma lesions. WB-CT scanning has a higher detection rate of lytic lesions compared with WBX but, in some studies, lesions in the skull and ribs were less well-detected with WB-CT, while other studies suggested a better detection rate with WB-CT [9, 10]. An other advantage of WB-CT is the fact that radiation exposure is comparable with WBX, and no intravenous contrast is needed [10].

MRI is used for detection of spinal cord compression and to differentiate myeloma from non-myeloma vertebral fractures. MRI has a high detecting rate of bone marrow involvement. MRI provides the opportunity to visualize bone marrow infiltration rather than defining osteolytic lesions. Therefore, in newly diagnosed patients, MRI may be less helpful since it detects bone lesions earlier than the myeloma-related bone destruction has occurred. In case of monoclonal gammapathy of unknown significance (MGUS), asymptomatic MM or solitary plasmacytoma of the bone, MRI can be used to distinguish a high-risk patient for developing symptomatic MM. Patients with more than one focal lesion on the MRI are classified as high risk for development of MM [10, 11].

An alternative manner of imaging MM activity is to target cellular properties of MM cells or micro-environment which can be accomplished by using different radio-labeled compounds to visualize the affected skeleton areas.

## Target mechanism in nuclear imaging

**Fig. 1:**
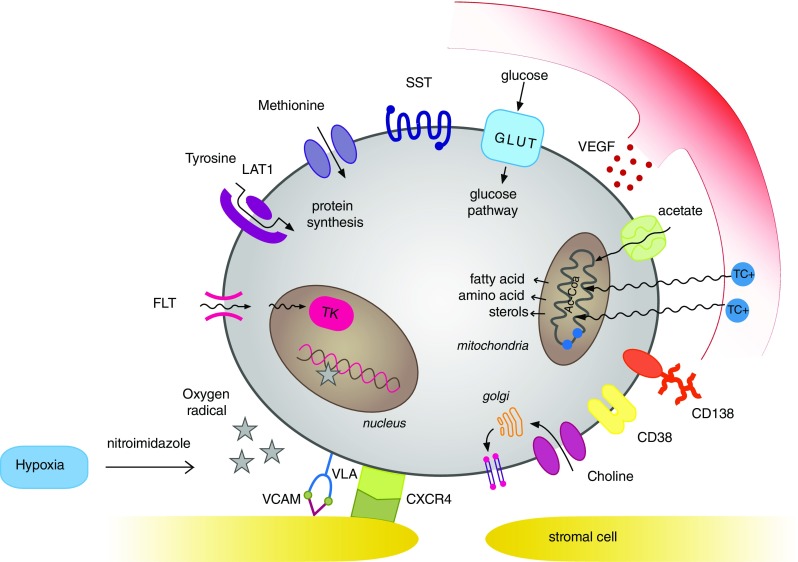
ᅟ

PET imaging is now widely used for the detection and follow-up of malignant disorders. In MM, PET tracers are used especially for detecting medullary and extramedullary disease. These nuclear tracers can also provide information about the degree of uptake by the lesions of interest, which is indicated by calculation of the standardized uptake value (SUV). In addition, PET scanning has shown promise for monitoring treatment response, since increase or decrease can be visualized and/or calculated compared to a baseline scan [12]. Recently, various specific PET tracers have been developed which might be useful in the workup of patients with newly diagnosed and relapsing MM. In the present review, we evaluate several nuclear tracers and nuclear imaging techniques as defined by their primary target (shown in Table 1 and Fig. 1).

**Table 1:** Various nuclear imaging techniques [PET and single-photon emission computed tomography (SPECT)] and their targets

Mechanism of action	Tracer	Target
**Cell metabolism**		
*Glucose*	[18 F]-FDG	Glucose uptake
*Amino acid*	[11C]-MET	Methionine
	[18 F]-FAMT	L-type aminoacid transporter 1
*Nucleotide*	[18 F]-FLT	Activity of thymidine kinase
	[11C] -4DST	Activity of thymidine kinase
*Membrane metabolism*	[11C]-ACT	Acetate/fatty acid synthesis
	[11C]-choline	Choline
**Receptor targeting**		
*Somatostatin receptor scintigraphy*	[111In]-pentetreotide	Somatostatin receptor
*Chemokine receptor 4*	[68Ga]-Pentixafor	CXCR-4 receptor
*Very-late-antigen-4*	[64Cu]-CB-TE1A1P-LLP2	VLA-4
**Mitochondrial activity**		
	[99mTc]-sestamibi	Mitochondria
	[99mTc]-tetrofosmin	Mitochondria
**Angiogenesis and hypoxia**		
*Hypoxia*	[18 F]-FAZA	Hypoxia
*Angiogenesis*	[89Zr]-bevacizumab	Circulating VEGF

Legend: [18 F]-FDG: [18 F]-fluorodeoxyglucose, [11C]-MET: [11C]-Methionine, [18 F]-FAMT: [18 F]-alpha-methyltyrosine, [18 F]-FLT: [18 F]-fluoro-3-deoxy-L-thymidine, [11C]-4DST: Methyl-11C-40- thiothymidine, [11C]-ACT: [11C]-acetate, [18 F]-FAZA: 1-α-D: −(5-deoxy-5-[18 F]-fluoroarabinofuranosyl)-2-nitroimidazole, VEGF: vascular endothelial growth factor, CXCR4: chemokine receptor 4, VLA-4: very late antigen-4

### Cell metabolism

#### Glucose

[18 F]-fluorodeoxyglucose PET ([18 F]-FDG-PET) uses enhanced glucose metabolic activity to visualize areas of interest [13]. [18 F]-FDG, a glucose analogue, is actively transported into cells mediated by a group of structurally related glucose transporter proteins (GLUT). Tumor cells, including MM cells, show increased numbers of these glucose transporters, particularly GLUT-1 and GLUT-3 [14]. Glucose and [18 F]-FDG are phosphorylated intracellularly by hexokinase. In contrast to glucose, [18 F]-FDG undergoes no further metabolism in the glucose pathway and becomes trapped intracellularly as [18 F]-FDG-6-phosphate [15]. The use of [18 F]-FDG-PET as a baseline scan has been studied extensively in newly diagnosed MM patients. Based on the number of focal lesions and the maximum SUV (SUV_max_), [18 F]-FDG-PET detects more lesions compared to WBX [9]. In addition, follow-up [18 F]-FDG-PET scans can be used to monitor treatment response and are of prognostic value for survival [12]. Complete normalization of the [18 F]-FDG-PET scan before and after ASCT correlates with both improved progression-free survival (PFS) and overall survival (OS) [12]. This imaging technique may also be useful in patients with relapsing MM since it is not hampered by the presence of pre-existing skeletal defects due to it visualizing areas of enhanced metabolic activity [9, 16–19]. In relapsing MM patients, only a few studies have been performed with [18 F]-FDG-PET [14, 20–22]. Disease activity is shown on the [18 F]-FDG-PET (Table 2A) in the majority of MM patients. A significantly higher number of positive lesions has been detected relative to WBX [14]. In about 25 % of the patients with relapsing disease, no defects on [18 F]-FDG-PET scans are shown, despite the presence of active disease. Comparable results have been reported in newly diagnosed MM patients, with 24 % of the patients having a negative [18 F]-FDG-PET scan (Table 2A) [12]. This is probably due to a diffuse distribution of malignant plasma cells in the skeleton. Skull lesions smaller than 1 cm are especially difficult to detect with [18 F]-FDG-PET [19] because of the high physiological uptake in the brain tissue.

**Table 2:** [18 F]-FDG-PET in MM compared to various nuclear imaging techniques (PET and SPECT)

**A**				
**Study**	**Tracer**	**n**	**MM**	**% pos [18 F]-FDG-PET**
*De Waal 2015*	[18 F]-FDG	44	R	82 %
*Lapa 2014*	[18 F]-FDG	37	R	76 %
*Zamagni 2011*	[18 F]-FDG	192	ND	76 %
*McDonald 2016*	[18 F]-FDG	192	ND	68 %
*B*				
**Study**	**Tracer**	**n**	**MM**	**pos scan vs pos [18 F]-FDG-PET (%)**
*Okasaki 2015*	[11C]-MET	10	R	100 % vs 60 %
*Lapa 2016*	[11C]-MET	43	32 R	91 % vs 77 %
*Isoda 2012*	[18 F]-FAMT	11	R	73 % vs 73 %
*Agool A 2006*	[18 F]-FLT	2	R	very low uptake
*Okasaki 2015*	11C] -4DST	10	R	80 % vs 60 %
*Lin C 2014*	[11C]-ACT	15	ND	86 % vs 67 %
*Nanni C 2007*	11C]-Choline	4	R	100 % vs 100 %
*Cassou-Mounat 2016*	11C]-Choline	21	R	71 % vs 71 %
*De Waal 2012*	111In]-Pentetreotide	18	R	52 % vs 71 %
*Philipp-Abbrederis K 2015*	68Ga]-Pentixafor	14	R	71 % vs 64 %
*Fonti R 2015*	99mTc]-Sestamibi	27	ND	89 % vs 97 %
*De Waal 2015*	[18 F]-FAZA	5	R	negative scan
*De Waal, unpublised*	89Zr]-Bevacizumab	5	R	neagtive scan

Legend A: [18 F]-FDG-PET in relapsing MM. B: Studies with different nuclear imaging techniques (PET and SPECT) in MM patients compared to [18 F]-FDG-PET before treatment was started.

[18 F]-FDG: [18 F]-fluorodeoxyglucose, MM: multiple myeloma, [11C]-MET: [11C]-methionine, [18 F]-FAMT: [18 F]-alpha-methyltyrosine, [18 F]-FLT: [18 F]-fluoro-3-deoxy-L-thymidine, [11C] -4DST: methyl-11C-40-thiothymidine, [11C]-ACT: [11C]-acetate, [18 F]-FAZA: 1-α-D: −(5-deoxy-5-[18 F]-fluoroarabinofuranosyl)-2-nitroimidazole; N: total number of patients studied; R: relapsed MM, ND: newly diagnosed MM.

Improvements have been made regarding uptake measurements with [18 F]-FDG-PET. Total lesion glycolysis (TLG) and metabolic tumor volume (MTV) can be calculated from [18 F]-FDG-PET and might be a more accurate measurement than the conventional measurements predicting overall tumor burden of focal lesions in MM. In a recent study of newly diagnosed MM patients, [18 F]-FDG-PET was performed at baseline (Table 2A). In this study, a TLG >620 g and MTV >210 cm [3] was found to be a significant predictor for a poorer PFS and OS [23]. Combining TLG and MTV with other risk factors like gene expression profiling and International Staging System (ISS) stage resulted in the identification of a high-risk subgroup [23].

The Italian group presented guidelines for uniforming [18 F]-FDG-PET quantification by using a five-point scale including: the metabolic state of the bone marrow, number and site of focal [18 F]-FDG-PET-positive lesions with or without osteolytic characteristics, presence and site of extramedullary disease, presence of paramedullary disease (a bone lesion involving surrounding soft tissues with bone cortical interruption), and presence of fractures. The visual degree of uptake is defined for the target lesion and extramedullary lesions according to the Deauville criteria [24]. In a small series, this five-point scale seemed reproducible and can be used for comparing [18 F]-FDG-PET scans from baseline to scans after treatment; furthermore, it provides a guideline to uniform research data [24].

#### Amino acids

Methionine, an amino acid required for protein synthesis, can be used for PET scanning. [11C]-Methionine ([11C]-MET) is frequently used for imaging brain tumours, since the physiological background uptake of [11C]-MET is low in the brain. Due to the active protein synthesis by the malignant plasma cells, this might also be a useful tracer for MM. In several studies, [11C]-MET was compared with [18 F]-FDG-PET (Table 2B) [25, 26]. Compared to [18 F]-FDG-PET, more lesions were detected with [11C]-MET, especially when a low number of aberrant plasma cells were present in the bone marrow (<30 %) [25]. A limitation for widespread use of [11C]-MET is the short half-life of approximately 20 minutes, necessitating production by an on-site cyclotron.

The amino acid transporter L-type amino acid transporter 1 (LAT-1) is overexpressed in a number of tumors including MM. LAT-1 provides a transporter function for protein synthesis. The amino acid tracer [18 F]-alpha-methyltyrosine ([18 F]-FAMT) is also transported by LAT-1. The uptake of this tracer correlates with LAT-1 expression [27]. In relapsing MM patients, a comparison has been made between [18 F]-FAMT and [18 F]-FDG-PET. A comparable number of lesions were detected with both imaging techniques, but the SUVmax was significantly lower in FAMT-PET compared to [18 F]-FDG-PET (Table 2) [27].

#### DNA proliferation marker

Thymidine, a DNA nucleoside required for DNA synthesis, can also be used as a tracer. Fluorothymidine is an analogue of the nucleoside thymidine (deoxythymidine) and can be labelled to produce [18 F]-fluoro-3-deoxy-L-thymidine ([18 F]-FLT). FLT is transported into the cell and is a substrate for thymidine kinase-1 (TK1), which is related to DNA synthesis and is, therefore, a surrogate marker for cell proliferation [28]. Using [18 F]-FLT PET scanning for solid malignancy, a high bone marrow background activity has been demonstrated, which indicates the proliferative activity of the hematopoietic cells in the bone marrow cavity [29]. Patients with MM were included in a pilot study using [18 F]-FLT with haematological malignancies. Areas with skeletal lesions showed a very low uptake of [18 F]-FLT compared to non-affected areas, which is in line with the low proliferative activity of MM cells [30]. Follow up scans with this tracer may be difficult since it has been shown that bone marrow cells undergo a phenotypic shift after chemotherapy and autologous stem cell transplantation, thereby altering their cycling time and the uptake seen on the [18 F]-FLT-PET scan [31]. The new tracer methyl-[11C]-40-thiothymidine ([11C]-4DST) has shown more positive findings. [11C]-4DST is more stable than [18 F]-FLT. Once [11C]-4DST is incorporated into DNA, de-phosphorylation occurs relatively rarely, unlike [18 F]-FLT. This has also been shown in a study of MM patients in which [11C]-4DST showed more positive findings per patient than [18 F]-FDG-PET, particularly in patients with low numbers of bone marrow plasma cells (Table 2B) [25].

#### Membrane

Acetate is an important metabolite that is used to synthesize amino acids, nucleotides and fatty acids. When ligated to coenzyme A (acetyl-CoA), it can be converted into fatty acids [32]. [11C]-acetate ([11C]-ACT) can be, like the natural precursor acetate, converted into fatty acids. In cell lines, it has been demonstrated that MM cells have a higher metabolic activity involving free fatty acids [28]. In newly diagnosed MM patients, [11C]-ACT PET identified more focal lesions than [18 F]-FDG PET (Table 2B) [33]. This result is promising, but needs to be verified in a larger number of patients, including relapsing MM patients. An additional player in fatty acid metabolism is choline, which is phosphorylated by choline kinase and incorporated into various phospholipids. In prostate cancer, [11C] or [18 F]-choline has frequently been used for monitoring disease activity. In two studies with [11C]-choline PET scanning in MM patients, the number of positive scans were similar when [11C]-choline was compared with [18 F]-FDG-PET, but more focal lesions were detected with [11C]-choline than with [18 F]-FDG-PET) [34, 35].

Cluster of differentiation 38 (CD38), which is a glycoprotein functioning in cell adhesion, signal transduction and calcium signaling [36], is highly expressed on myeloma cells, but at relatively low levels on normal hematopoietic cells and in some tissues of non-hematopoietic origin, like lung tissue. Currently, an anti-CD38 human monoclonal antibody, daratumab, has been developed. This drug is promising in the treatment of MM. Treatment with daratumumab monotherapy in relapsed and refractory MM patients did have a significant effect on PFS [36]. Phase 3 trials combining daratumumab with conventional treatments such as bortezomib and lenalidomide are ongoing. Use of radio-labeled anti-CD38 could be a promising approach not only for diagnostic purposes and treatment monitoring, but it might also be used in the context of radio-immunotherapy due to the restricted expression of CD38 on normal tissues. Preclinical studies have already been performed in xenograft mice models with radio-immunoconjugates consisting of the α-emitter [213Bi] coupled to the anti-CD38 monoclonal antibody, with a significant targeting of CD38 [37].

CD138, or syndecan-1, is a member of the syndecan family, expressed by epithelial cells, precursor B cells, and normal plasma cells. CD138 is highly expressed on myeloma cells. Phase I–II studies have been initiated with anti-CD138, called Indatuximab [38, 39]. Alpha-radio-immunotherapy treatment using a [213Bi]-labelled anti-mouse CD138 antibody has been performed in mouse models, showing promising results. Mice treated with [213Bi]-CD138 had a longer median survival than the control group [40].

### Receptor targeting

In vitro studies with plasma cell lines have shown that somatostatin receptors, especially subtypes 1, 2 and 5, are highly expressed on MM cells [41]. Somatostatin receptor scintigraphy (SRS) using the single-photon emission computed tomography (SPECT) tracer [111In]-pentetreotide is able to visualize somatostatin receptors, especially subtypes 2 and 5 [42]. SPECT has, by definition, a lower spatial resolution and less quantification possibilities, compared to PET. Studies with SRS in MM patients have been performed specifically in relapsing MM patients. A positive SRS scan was reported in 83 % of the patients, which is significantly higher than the results obtained with WBX [43, 44]. However, when SRS was compared to [18 F]-FDG-PET, the latter identified significantly more focal lesions [44], which may be explained by the better resolution of PET compared to SPECT. SRS is also used in the workup of neuroendocrine tumors (NET). In this context, [68Ga]-DOTA-TOC/TATE/NOC/lanreotide PET/CT is used, which has superior resolution and, hence, better sensitivity than the conventional SRS, thereby replacing SRS for staging NET [45]. Considering these properties, [68Ga]-DOTA subtypes might also be useful for diagnostic purposes in MM.

Chemokine receptor 4 (CXCR4) is expressed on hematopoietic stem and progenitor cells residing in the bone marrow niche, which has an important function in the homing of these cells to the bone marrow compartment. Disrupting this interaction by Plerixafor, a CXCR4 antagonist, results in mobilization of stem cells out of the bone marrow niche, a property used for stem cell mobilization. CXCR4 is also expressed on other cell types of the hematopoietic system, including plasma cells. Approximately 50 % of the MM cells express CXCR4 in high density [46]. [68Ga]-pentixafor is a labelled peptide with high affinity for CXCR4, which has been studied in relapsing MM patients in comparison to [18 F]-FDG-PET. The results showed that a high number of the MM patients have a positive [68Ga]-pentixafor scan [46] (Table 2B). Due to these promising results, the anti-CXCR4 antibody has been labeled with [177Lu]-pentixather or [90Y]-pentixather with the aim of performing radio-immunotherapy. Recently, three heavily pretreated myeloma patients were treated with [177Lu]-pentixather or [90Y]-pentixather after CXCR4 expression was confirmed with a [68Ga]-pentixafor PET scan. Two patients responded to treatment, showing a decrease in free-light chain ratio and a reduction of SUVmax on the [18 F]-FDG-PET scan. The third patient died due to sepsis 3 weeks after [177Lu]-pentixather therapy. PFS was short, and ranged between 3 and 6 months [47]. Based on these pilot studies, [68Ga]-pentixafor scanning might be used to select patients treatment with radiolabeled [68Ga]-pentixafor and to monitor the effects of therapy [47].

Another membrane receptor is the very late antigen-4 (VLA-4, α4β1integrin, CD49d/CD29), which is a transmembrane adhesion receptor. VLA-4 is over-expressed on MM cells and plays a key role in the adhesion and spreading through the bone marrow compartment. VLA-4 binds to vascular cell adhesion molecule-1 (VCAM-1) and fibronectin of bone marrow stromal cells. The PET tracer [64Cu]-CB-TE1A1P-LLP2 is now under investigation in preclinical models [48].

### Mitochondrial activity

[99mTc]-sestamibi and [99mTc]-tetrofosmin have been developed for myocardial perfusion SPECT imaging. These compounds consist of a lipophilic monovalent cation, which is sequestered in the mitochondria by the large negative membrane potential [28]. [99mTc]-sestamibi SPECT has also been studied in MM and compared with [18 F]-FDG-PET and MRI. In newly diagnosed MM patients, [18 F]-FDG-PET detected more focal lesions than [99mTc]-sestamibi (Table 2B) [49], probably due to better spatial resolution and different target imaging. Furthermore, [18 F]-FDG-PET and [99mTc]-sestamibi detected more lesions, including extramedullary disease, than MRI [49].

### Angiogenesis & hypoxia

**Fig. 2:**
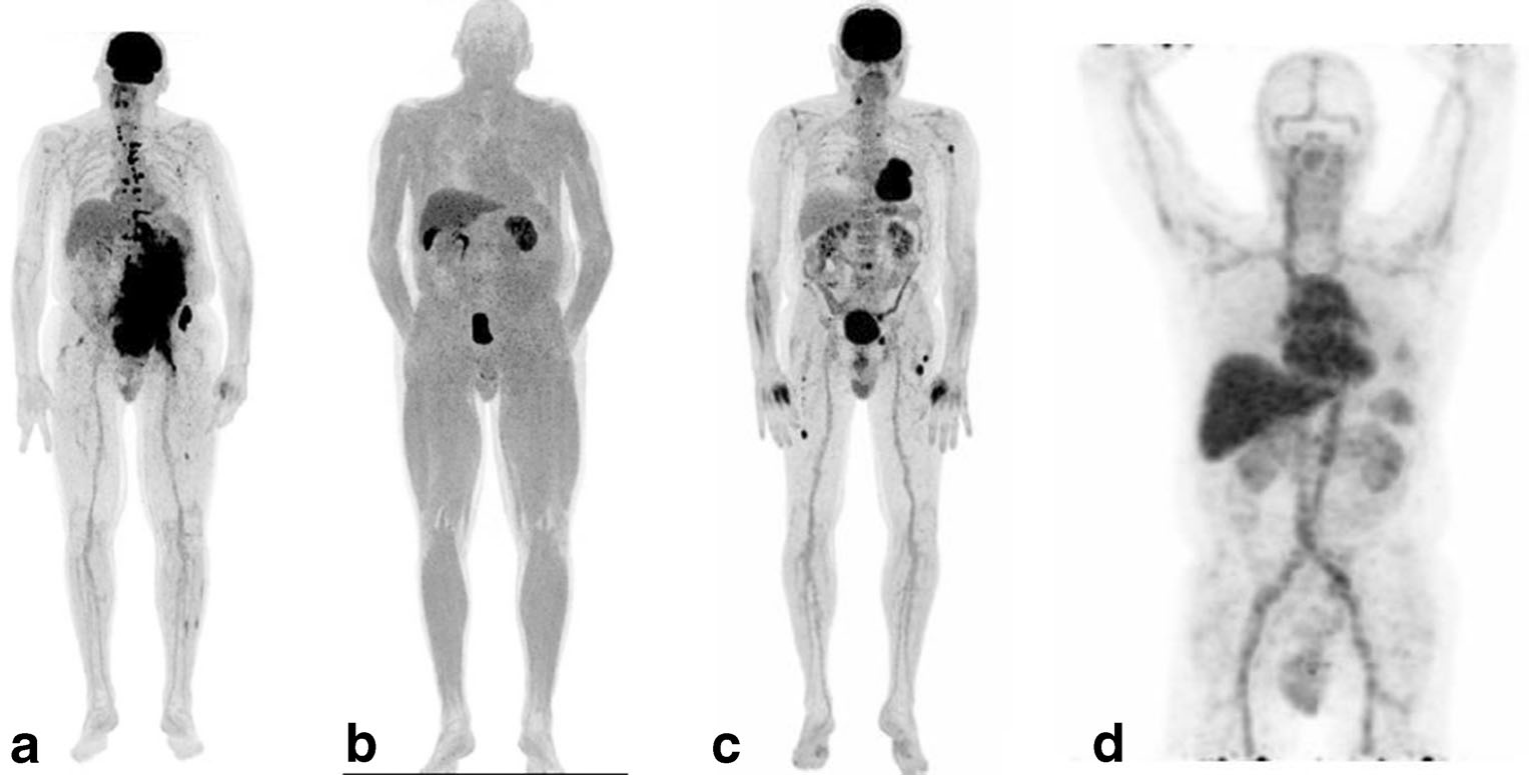
Various PET imaging in MM patients. Legend: **a**. [18 F]-FDG-PET with diffuse hotspots in the skeleton and extramedullary. **b**. [18 F]-FAZA of the same patient as *a* with no hot spots. **c**. [18 F]-FDG-PET, several bone hot spots. **d**. [89Zr]-Bevacizumab of the same patient as *c* with no hot spot in the skeleton

The increased FDG uptake by malignant MM cells is related to higher metabolic activity. This might be a consequence of tumour hypoxia resulting from increased O_2_ consumption by the malignant MM cells or due to altered micro-vascularisation of the bone marrow containing plasma cells. Due to hypoxia, hypoxia inducible factor (HIF)-1α and HIF-2α are produced by the malignant cells or by the surrounding endothelial cells, which triggers the production of vascular endothelial growth factor (VEGF), resulting in increased micro-vessel density (MVD) around the malignant plasma cells. Several studies have shown that increased MVD correlates with tumor progression [50–53]. A relatively low MVD has been observed in smouldering MM, which increases significantly during disease progression [51]. The PET tracer 1-α-D: −(5-deoxy-5-[18F]-fluoroarabinofuranosyl)-2-nitroimidazole (18F-FAZA), has been developed for visualizing in vivo tumor hypoxia. Under hypoxic conditions, two nitroimidazole compounds undergo reduction, forming highly reactive oxygen radicals and binding to macromolecules inside the cell. [18F]-FAZA has been used in patients with solid tumors, such as head and neck cancers and non-small cell lung cancers [54, 55]. At our institute, five patients diagnosed with relapsing MM based on a positive [18 F]-FDG-PET scan underwent [18 F]-FAZA scanning. However, enhanced uptake of [18 F]-FAZA was not demonstrated in any of the patients, despite the presence of focal disease (Fig. 2a and b) [14]. These findings suggest that the degree of hypoxia is not substantially different in MM spots compared to the surrounding bone marrow compartment. An alternative approach might be to use tracers that bind to the VEGF produced by MM cells. This can be achieved by labeling bevacizumab, a recombinant, humanised monoclonal antibody that binds with high affinity to all isoforms of free human VEGF. Treatment with bevacizumab is well-established in solid tumors such as colon cancers and renal cell carcinomas. [89]Zr-bevacizumab PET scanning has been used to detect solid tumors like breast cancer and demonstrated positive scans in 96 % of the patients [56]. [89Zr]-Bevacizumab PET scanning was also performed in five relapsed MM patients (Fig. 2c and d). All the patients had clinical progression of MM, and positive lesions were detected on [18 F]-FDG-PET. Unfortunately, no lesions were detected with [89Zr]-bevacizumab PET scanning in any of the patients.

## Discussion

Various nuclear imaging techniques and tracers have been used for detecting disease activity and bone lesions in newly diagnosed MM patients, but few studies have been conducted in relapsed MM patients. Nuclear imaging can be helpful, especially in relapsed MM because it is frequently difficult to detect new disease activity with WBX, MRI or bone marrow biopsy due to its scattered growth pattern. Additionally, nuclear imaging can be helpful in determining the response to treatment, which might have also prognostic significance for clinical outcome. As shown in Table 1 and Fig. 1, the tracers used for nuclear imaging target different aspects of the cellular properties of the plasma cells. Most studies have been performed with [18 F]-FDG-PET. [18 F]-FDG-PET is widely used and available for patient care. In newly diagnosed MM, [18 F]-FDG-PET detects more lesions, including extramedullary disease, compared to WBX and provides useful information about treatment response and outcome. Currently, WB-CT-scan is replacing WBX as standard care for the detection of skeleton lesions. The use of nuclear tracers provides additional information, especially regarding the metabolic status of the disease, and can be used for treatment monitoring. In newly diagnosed MM patients, it has been shown that complete normalization of the [18 F]-FDG-PET scan before and after ASCT correlates with improved PFS and OS [12]. For relapsing MM patients, similar results have been reported in the diagnostic setting, but no conclusive data are available on treatment outcome, which might be related to the small number of patients studied. In about 25 % of the patients with relapsing MM, no defects are shown on [18 F]-FDG-PET scans despite the presence of active disease (Table 2A). Comparable results have been reported in newly diagnosed MM patients, indicating that a negative [18 F]-FDG-PET scan does not exclude disease activity. Various other tracers can be used to visualize MM activity, as shown in Table 2. [11C]-Methionine, [11C]-acetate and [11C]-choline are promising tracers for detecting skeleton lesions. A drawback of these tracers is the necessity of an on-site cyclotron, which prevents wide distribution since it has to be applied shortly following production. [18 F]-Choline, has a longer half-life, is commercially available, and could be a good alternative.

Angiogenesis plays a distinct role in disease progression in MM [50–52]. Therefore, it seems promising to use tracers related to this process. Immunohistochemical staining of bone marrow biopsies of MM patients show increased MVD, increased VEGF expression by plasma cells and increased expression of HIF-2α by the endothelial cells. However, no positive scans were obtained in proof of principle work, neither with [18 F]-FAZA scanning nor with [89Zr]-bevacizumab PET scanning, despite strongly positive results with [18 F]-FDG-PET scanning. It has been suggested that HIF-1α increases immediately in response to hypoxia, whereas HIF-2α responds to prolonged periods of hypoxia [57]. The negative results might be related to technical or biological issues, for example, due the fact that the bone marrow itself is in a state of low hypoxia, which might hamper detection by [18 F]-FAZA.

With immunohistochemical staining we detected high levels of intracellular VEGFa and clear alteration in the microvasculature, suggesting that the observed VEGF is secreted by the malignant plasma cells. However, this VEGFa could not be visualized by [89Zr]–bevacizumab. This is probably due to the limited concentration resulting from rapid binding to plasma cells or platelets and the limitations in spatial resolution with the use of 89Zr-tracers. Based on these first findings, both [18 F]-FAZA and [89Zr]-bevacizumab do not appear to be useful in the work-up of relapsing MM patients.

### Future perspectives

Stand-alone MRI is frequently used in the diagnosis of MM, especially for the spine and pelvis. PET/MRI appears to be a promising new technique for diagnosis and follow up of myeloma patients. It is now possible to perform PET/MRI studies in which metabolic components are combined with the anatomic components of the MRI. In MM patients, [18 F]-FDG-PET/CT has been compared to FDG-PET/MRI. Almost all lesions detected by [18 F]-FDG-PET/CT were also detected by FDG-PET/MRI [58]. Further investigation and optimalization of the protocol for PET/MRI is needed to provide more information about the role of PET/MRI for diagnostic purposes and response monitoring in MM. Novel MRI sequences are now available. For example, diffusion weighted imaging (DWI) and delayed contrast enhancement (DCE) seem to improve the diagnostic properties of MRI in MM patients [59, 60]. Combining PET with these novel MRI techniques may be valuable for optimal diagnosis and evaluation of relapsed MM activity.

The tracers discussed in this review might also be useful in the radio-immunotherapy setting, as described for [68Ga]-pentixafor. However, a drawback might be the negative effects on normal hematopoietic cells due to the high expression of CXCR4 on these cells. These negative effects on normal hematopoietic cells might be circumvented if this type of radio-immunotherapy is used in the context of autologous stem cell transplantation.

In the future, radiolabeled daratumumab might be very interesting; preclinical data is promising and treatment with daratumumab is well-tolerated with encouraging results [36, 37]. Also, anti-CD138 might be a promising approach [38]. CD319 also called signaling lymphocytic activation molecule (SLAM) F7 is a receptor present on immune cells including plasma cells. The antibody against SLAMF7 is called elotuzumab. Treatment with this antibody demonstrates promising results in relapsing MM patients [39]. Like anti-CD38 and anti-CD138, labeling of this antibody might provide disease information and perhaps to be a new target for radio-immunotherapy. Furthermore, labeling with [64Cu]-chelates might also be used in future research. A mice study with [64Cu]-anti-CD138 showed promising results [61].

Nuclear imaging is of growing importance in the diagnostic process and follow up of MM patients. It provides important information regarding the anatomical localization and the metabolic activity of the areas of interest. Combining both pieces of information according to uniform scoring systems will provide important information for the individual patient but also for the comparison of future study protocols. Examples are the five-point scaling score shown by the Italian group [24] and the combined use of TLG and MTV [23].

In conclusion, nuclear imaging is an important tool for diagnostic purposes and response monitoring, particularly in relapsing MM patients. Various nuclear tracers have been developed to detect bone, bone marrow and extramedullary involvement. In the future, these tracers might also be used for treatment monitoring, including detection of minimal residual disease.
